# Lithospermic acid attenuates 1-methyl-4-phenylpyridine-induced neurotoxicity by blocking neuronal apoptotic and neuroinflammatory pathways

**DOI:** 10.1186/s12929-015-0146-y

**Published:** 2015-05-28

**Authors:** Yun-Lian Lin, Huey-Jen Tsay, Tzu-Hsuan Lai, Tsai-Teng Tzeng, Young-Ji Shiao

**Affiliations:** National Research Institute of Chinese Medicine, 11221 Taipei, Taiwan, Republic of China; Institute of Neuroscience, Brain Research Center, National Yang-Ming University, 11221 Taipei, Taiwan Republic of China; Institute of Biopharmaceutical Science, National Yang-Ming University, 11221 Taipei, Taiwan Republic of China; Ph.D Program for the Clinical Drug Discovery from Botanical Herbs, College of Pharmacy, Taipei Medical University, 110 Taipei, Taiwan Republic of China

**Keywords:** Lithospermic acid, CATH.a cells, ICR mice, Substantia nigra ,1-methyl-4-phenylpyridin, Parkinson’s disease

## Abstract

**Background:**

Parkinson’s disease is the second most common neurodegenerative disorders after Alzheimer’s disease. The main cause of the disease is the massive degeneration of dopaminergic neurons in the substantia nigra. Neuronal apoptosis and neuroinflammation are thought to be the key contributors to the neuronal degeneration.

**Results:**

Both CATH.a cells and ICR mice were treated with 1-methyl-4-phenylpyridin (MPP^+^) to induce neurotoxicity *in vitro* and *in vivo*. Western blotting and immunohistochemistry were also used to analyse neurotoxicity, neuroinflammation and aberrant neurogenesis *in vivo*. The experiment in CATH.a cells showed that the treatment of MPP^+^ impaired intake of cell membrane and activated caspase system, suggesting that the neurotoxic mechanisms of MPP^+^ might include both necrosis and apoptosis. Pretreatment of lithospermic acid might prevent these toxicities. Lithospermic acid possesses specific inhibitory effect on caspase 3. In mitochondria, MPP^+^ caused mitochondrial depolarization and induced endoplasmic reticulum stress via increasing expression of chaperone protein, GRP-78. All the effects mentioned above were reduced by lithospermic acid. In animal model, the immunohistochemistry of mice brain sections revealed that MPP^+^ decreased the amount of dopaminergic neurons, enhanced microglia activation, promoted astrogliosis in both substantia nigra and hippocampus, and MPP^+^ provoked the aberrant neurogenesis in hippocampus. Lithospermic acid significantly attenuates all of these effects induced by MPP^+^.

**Conclusions:**

Lithospermic acid is a potential candidate drug for the novel therapeutic intervention on Parkinson’s disease.

## Background

Parkinson’s disease (PD) is characterized by the selective loss of dopaminergic neurons and neuroinflammation in the substantia nigra (SN) [1–3]. Even after numerous studies, the cause of dopaminergic cell degeneration in SN of PD patients still has not been identified with certainty. Therefore, several experimental models for the therapeutic research on PD have been developed to investigate the pathogenesis and pathophysiology of PD [4, 5].

MPTP (1-methyl-4-phenyl-1,2,3,6-tetrahydropyridine), a neurotoxin that can induce symptoms similar to those observed in PD patients [6] and induces selective loss of dopaminergic neurons in SN of mice [4, 5]. MPTP must be converted into 1-methyl-4- phenylpyridine (MPP^+^) in the glial cells and then taken up by dopaminergic neurons via the dopamine transporter. Consequently, MPP^+^ is concentrated inside the mitochondria where it potently inhibits complex I of the electron transport system [7]. This result in ATP depletion, loss of mitochondrial membrane potential, and the formation of reactive oxygen species (ROS) and reactive nitrogen species (RNS), activation of caspase-9 and −3 [8], increasing intracellular Ca^2+^ concentrations [9], enhancing endoplasmic reticulum (ER) stress [10, 11], and results in programmed cell death.

Several lines of evidence suggest that apoptotic cell death of dopaminergic neurons in PD is driven in part by neuroinflammation [12, 13]. The production of toxic levels of ROS and RNS by activated microglia is believed to be a major threat for dopaminergic neurons survival [12]. Persistent microglial activation has been detected in the SN of humans and mice [14, 15]. Moreover, the short-term MPTP induced a significant increase in the numbers of newborn type1 and type2a neurons which represent a subpopulation of rarely dividing cells with glial characteristics, which are suggested to give rise to immature neurons in the course of adult neurogenesis [16].

A variety of polyphenolic compounds has been isolated from *Tournefortia sarmentosa* Lam. (Boraginaceae) (Chinese name: Teng Zi Dan), widely used in Taiwan as a detoxicant and anti-inflammatory agent [17]. Most of these polyphenolic compounds possess antioxidative activity, including tournefolic acid B (TAB), TAB methyl ester (TABM), and lithospermic acid (LSA). The TAB and TABM exhibit neuroprotective potency against glutamate-, NMDA-, and Aβ-mediated neurotoxicity [18–20]. LSA was also extracted from *Salvia miltiorrhiza* Bge (Labiatae) [21] and *Origanum vulgare* [22], and has been investigated to possesses the effects on anti-oxidation and anti-inflammation [23]; and anti-atherosclerosis [24].

In this study, we use both cell and animal models for PD. To study the MPP^+^-mediated neurotoxicity *in vitro*, murine dopaminergic neuron cell line (CATH.a) was employed according on their activity to synthesize abundant dopamine and norepinephrine and expressing the appropriate catecholaminergic biosynthetic enzymes, tyrosine hydroxylase (TH) and dopamine β-hydroxylase [25]. For comparison, primary cortical neurons, in which doparminergic neuron is not the major neuronal type, were also employed. On the other hand, to examine the MPP^+^-mediated neurotoxicity, neuroinflammation, and neurogenesis *in vivo*, intracerebral ventricle (icv)-injection of MPP^+^ in mice was used. The objective of this study was to investigate influence of LSA on MPP^+^-induced neurotoxicity alone and neurotoxicity with neuroinflammation and neurogenesis in cell and animal models, respectively. Our results demonstrated that LSA effectively reduced MPP^+^-induced neurotoxicity *in vitro* and *in vivo*. Furthermore, LSA effectively attenuated neuroinflammationm and the aberrant neurogenesis *in vivo*.

## Methods

### Cell cultures and treatments

CATH.a cells were cultured in RPMI 1640 medium containing 10 units/ml penicillin, 10 μg/ml streptomycin, 4 % fatal bovine serum and 6 % horse serum.

Primary cultures of neonatal cortical neurons were prepared from the cerebral cortex of Sprague Dawley rat pups at postnatal day 1 as described previously [26]. Briefly, pups were anesthetized with ether and sacrificed by decapitation. The cortex was digested in 0.5 mg/ml papain at 37 °C for 15 min and dissociated in Hibernate A medium (containing B27 supplement) by trituration. Cells were plated (5 × 10^4^ cells/cm^2^) onto poly-L-lysine-coated plates and maintained in Neurobasal medium containing B27 supplement, 10 units/ml penicillin, 10 μg/ml streptomycin, and 0.5 μg/ml glutamine (5 % CO_2_/9 % O_2_) for 3 days. Cells were then exposed to cytosine-β-D arabinofuranoside (5 μM) for 1 day to eliminate the proliferation of non-neuronal cells. The cells were used for the experiment on the fifth day.

CATH.a cells or cortical neurons were pre-treated with vehicle, LSA or TAB for 30 min and then MPP^+^ were added into the culture for inducing neurotoxicity.

### Measurement of cell viability

The calcein/ethidium homodimer-1 staining and the reduction of 3-[4,5-dimethylthiazol-2-yl]-2,5-diphenyl-tetrazolium bromide (MTT) were used to evaluate cell viability. Cells were loaded with 1 μM of both calcein AM and ethidium homodimer-1 at room temperature for 30 min. The cells were observed by laser-scanning confocal fluorescence microscope (Leica CS SP, Wetzlar, Germany; Zeiss LSM780, Carl Zeiss, Jena, Germany). Cells were incubated with 0.5 mg/ml MTT for 1 h. The formazan particles were dissolved with dimethyl sulfoxide (DMSO). OD600 nm was measured using an ELISA reader.

### Measurement of superoxide anion (O_2_^−^.)

The intracellular level of O_2_^−^. was measured by dihydroethidium assay as described previously [19]. Treated cells were loaded with 3.2 μM dihydroethidium at 37 °C for 30 min and then observed by a Leica DMIRB fluorescence microscope. The intensity of fluorescence in nuclear position was measured by using MetaMorph software (Universal Imaging Co., West Chester, PA).

### Determination of mitochondrial membrane potential

Cells were treated with MPP^+^ (300 μM) or MPP^+^ combined with LSA (100 μM) for 2 hours, and then cells were loaded with 100 nM Tetramethyl Rhodamine Methyl Ester (TMRM) at 37 °C for 20 min. After wash with Hanks’ balanced salt solution (HBSS; 137 mM NaCl, 5.4 mM KCl, 0.4 mM KH_2_PO_4_, 4.2 mM NaHCO_3_, 0.5 mM MgCl_2_, 0.6 mM MgSO_4_, and 5.6 mM D-glucose, pH 7.4), cells were incubated in HBSS containing 2 mM CaCl_2_. The fluorescence of TMRM was detected by a Leica DMIRB fluorescence microscope with an excitation wavelength of 555 nm.

### Immunoblots

For Western blot analysis, samples (6 μg protein) were separated by sodium dodecyl sulfate-polyacrylamide gel electrophoresis (15 % gels) and were then transferred to PVDF membranes. The primary antibodies used were as follows: mouse monoclonal antibody to actin and α-spectrin (Invitrogen), rabbit polyclonal antibody to caspase 3 (Millipore), cleaved caspase 3, caspase 7, cleaved caspase 7, caspase 9, cleaved caspase 9, caspase 12, cleaved poly ADP ribose polymerase (PARP), c-jun N-terminal kinase (JNK), pJNK(T183/Y185) (Cell signalling) and 78 kDa glucose-regulated protein (GRP-78) (Santa Cruz). The secondary antibodies were anti-rabbit IgG antibody conjugated with horseradish peroxidase (GE Healthcare) and anti-mouse IgG antibody conjugated with horseradish peroxidase (Jackson ImmunoResearch). Enhanced chemiluminescence detection reagents (GE Healthcare) were used for detection. Bands were quantified using Fujifilm LAS-3000 Luminescent Image Analyzer (Tokyo, Japan).

### Animals and treatment

The Institutional Animal Care and Use Committee at the National Research Institution of Chinese Medicine approved the animal protocol (IACUC No: 95-A-08). Six week-old male ICR mice were housed for 1 week under standard conditions at 25 ± 2 °C with a 12-h light/dark cycle and were allowed free access to water and standard chow. Administration (icv) of MPP^+^ (12 μg in 2 μl saline) was performed unilaterally on male ICR mice (8 weeks old). The mice were anesthetized with intraperitoneal (ip) chloral hydrate (Sigma; 0.4 g/kg body weight, maintained with 0.1 g/kg hourly) and fixed into a stereotaxic apparatus. The dorsal surface of the skull was exposed with a midline incision, and a burr hole was drilled at the following coordinates: anteroposterior, 0.22 mm caudal to bregma and 1 mm right lateral to midline. A 10-μl Hamilton syringe fitted with a 25-gauge needle was pre-loaded by 1 μl MPP^+^ solution (12 μg MPP^+^ in 1 μl saline) followed by 1 μl LSA solution (5 μg LSA in 1 μl saline). The injection syringe was placed over the burr hole and lowered 2.5 mm into the surface of the brain, and the solution was injected at a rate of 0.2 μl/min. The needle was then left in place for 2 min before it was slowly retracted. Control animals were injected with saline. MPP^+^ is injected into lateral ventricle to induce a Parkinson-like syndrome and lost of dopaminergic neurons in SNPc, and the proper dose is determined by trial test. The final amount of MPP injected was set at 12 μg MPP^+^.

### Tissue processing

At day 5 post MPP^+^ injection and vehicle injection, mice were deeply anesthetized with chloral hydrate and perfused through the heart with 30 ml of saline followed by 30 ml of fixative solution containing 4 % formaldehyde in saline. The brain was removed, post-fixed in 4 % formaldehyde for 18 h at 4 °C and cryoprotected in 30 % sucrose solution in 0.1 M phosphate-buffered saline (PBS). A cryostat was used to cut 30-μm coronal sections through the dorsal hippocampus, which were collected serially in PBS.

### Immunohistochemistry

Brain sections were incubated in blocking solution (PBS containing 5 % normal goat serum, 2 % Triton X-100, 0.02 % bovine serum albumin) overnight at 4 °C and left overnight at 4 °C in staining solution (PBS containing 5 % normal goat serum, 0.25 % Triton X-100, 0.02 % bovine serum albumin) with primary antibodies, including mouse monoclonal antibody to glial fibrillary acid protein (GFAP) and tyrosin hydrolase (TH) (Invitrogen); rabbit polyclonal antibody to doublecortin (Abcam); and mouse monoclonal antibody to ionized calcium-binding adaptor molecule-1 (Iba-1, Abcam). Sections were then incubated in staining solution containing Hoechst33258 (Invitrogen, 2 μg/ml), Fluorescein isothiocyanate-conjugated goat anti-mouse IgG and cy5-conjugated goat anti-rabbit IgG or cy5-conjugated goat anti-rabbit IgG (1:200; Jackson ImmunoResearch) in the dark overnight at 4 °C. Sections were then washed in PBS and mounted with Aqua Poly/Mount (Polyscience Inc., Warrington, PA, USA). The sliced tissues were examined using a laser-scanning confocal microscope (Zeiss LSM780; Carl Zeiss, Jena, Germany).

### Quantitative immunofluorescence analysis

All analyses were calculated within a field (250 × 250 μm^2^) of substantia nigra pars reticulata (SNpr), substantia nigra pars compacta (SNpc), Cornu Ammonis (CA)1 and dentate gyrus (DG). The expression of GFAP and Iba-1 expression was calculated from the immunoreactivity (IR) of each protein that displayed as the optical density (arbitrary unit) of each image.

### Statistic analysis

The results are expressed as the mean ± standard deviation (S.D.) and were analyzed by analysis of variance (ANOVA) with post hoc multiple comparisons corrected with Bonferroni tests.

## Results

### MPP^+^ mediates neurotoxicity in CATH.a cells and cortical neurons through distinct mechanisms

**Fig. 1:**
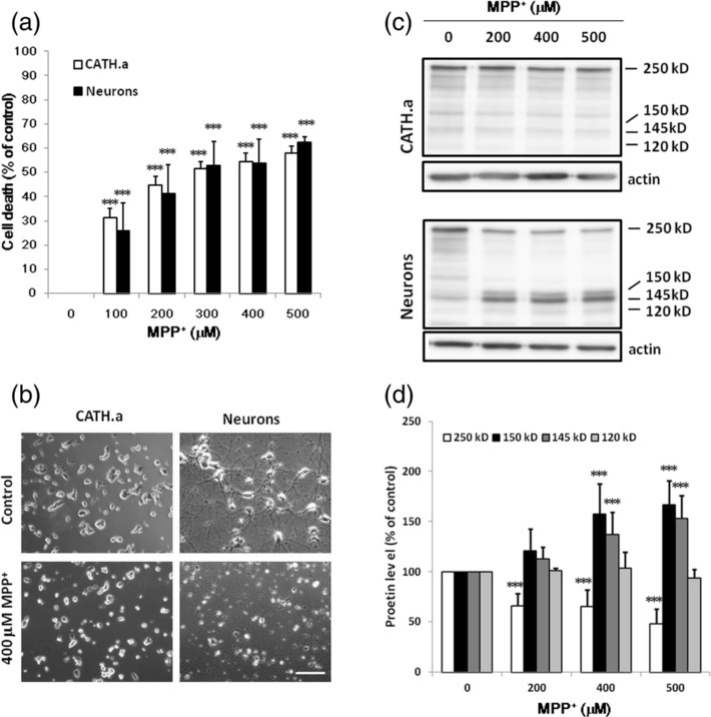
The Neurotoxicity of MPP^+^ on CATH.a cells and primary cortical neurons. **a**, **b** CATH.a cells and cortical neurons were cultured with various concentrations of MPP^+^ for 24 hours. The cell viability of CATH.a cells (opened bars) and cortical neurons (closed bars) were detected by MTT reduction assay (**a**). Results are means ± S.D. from three independent experiments. Significant differences between the cells treated with vehicle and MPP^+^ were indicated by ***, P < 0.001. The representative phase contrast morphology of the treated cells is shown in panel (**b**). Scale bar: 50 μm. The representative immunoblots of α-spectrin, spectrin degraded fragments and β-actin are shown in panel (**c**). In cortical neurons, the activation of calpain cleaved 250-kDa α-spectrin (opened bars) to produce 150- (closed bars) and 145-kDa (dark gray bars) fragments, whereas activation of caspase 3 generated 120- (light gray bars) and 150-kDa fragments of α-spectrin (**d**). Results are means ± S.D. from three independent experiments. Significant differences between the cells treated with vehicle and MPP^+^ were indicated by ***, P < 0.001

Treatment with MPP^+^ elicited cell death of CATH.a cells and cortical neurons in a concentration-dependent manner as determined by MTT reduction (Fig. 1a). MPP^+^ (300 μM) elicited cell death of CATH.a cells and cortical neurons by 51.65 ± 2.80 % and 52.66 ± 10.12 %, respectively. Morphologically, MPP^+^-provoked cell death of CATH.a cells and cortical neurons coincided with the appearance of cell atrophy and discontinuous neurites, respectively (Fig. 1b). Proteolytically, treatment with MPP^+^ induced cleavage of α-spectrin in cortical neurons but not in CATH.a cells. In cortical neurons, both the 150- and 145-kDa protein fragments (the activity of calpain), but not the 120-kDa fragment (the activity of caspase 3), were significantly appeared after the treatment of MPP^+^ (Fig. 1c, d).

### LSA protect CATH.a cells but not cortical neurons against MPP^+^-mediated neurotoxicity

**Fig. 2:**
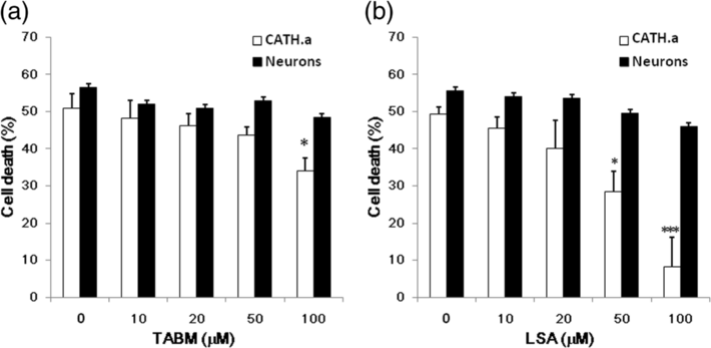
The effect of TABM and LSA on the Neurotoxicity of MPP^+^. CATH.a cells (opened bars) and cortical neurons (closed bars) were pretreated with various concentrations of TABM (**a**) or LSA (**b**) for 30 min, and then the cells were cultured with 250 μM MPP^+^ for 24 hours. Cell viability was mesured using MTT reduction assay. Results are means ± S.D. from three independent experiments. Significant differences between the cells treated with MPP^+^ combined with TABM or LSA were indicated by *, P < 0.05; ***, P < 0.001

The effects of TABM and LSA on MPP^+^-induced neurotoxicity were investigated. From Fig. 1a, the concentration of MPP^+^ to induce 50 % cell death is 250 μM. Therefore, in this assay 250 mM MPP^+^ were adopted. The structure of TABM and LSA were showed in Fig. 2a. TABM and LSA at 100 μM did not significantly alter the cell viability of both cells (Data not shown). To cortical neurons, both TABM and LSA fail to ameliorate the detrimental effect of MPP^+^ (Fig. 2b, c). To CATH.a cells, the MPP^+^-mediated cell death was 33.06 % attenuated by 100 μM TABM (Fig. 2b), and was significantly attenuated by LSA in a concentration-dependent manner. LSA at 50 and 100 μM abrogated MPP^+^-mediated CATH.a cell death by 42.29 % and 83.19 %, respectively (Fig. 2c).

**Fig. 3:**
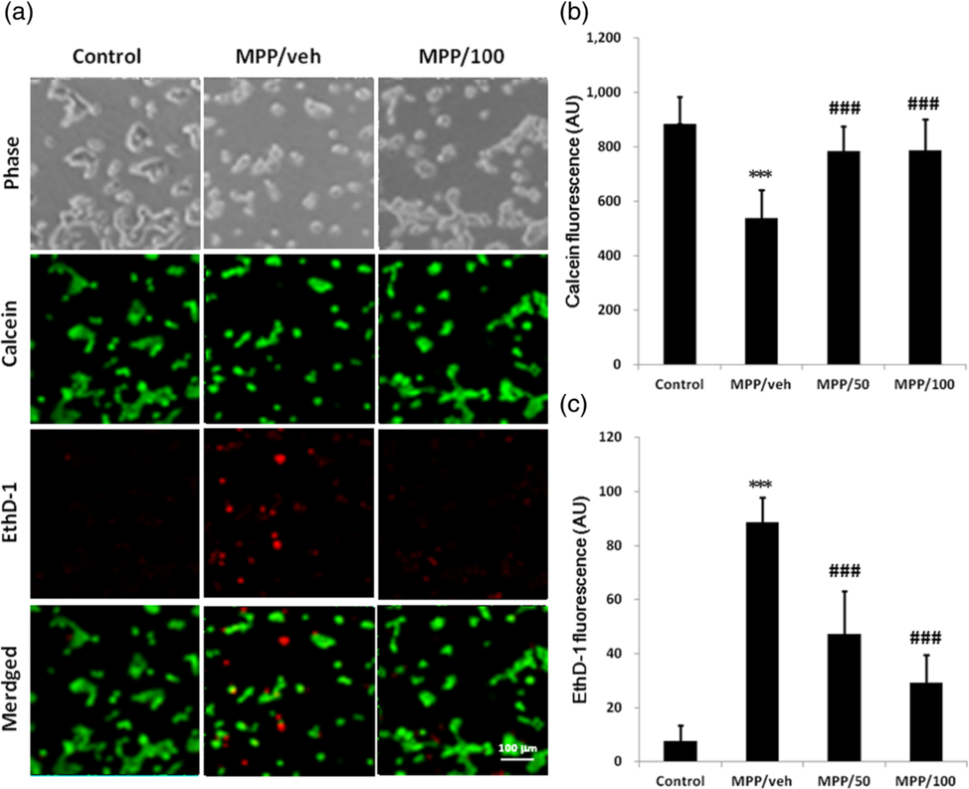
The Neurotoxicity of MPP^+^ and neuroprotective effect of LSA in CATH.a cells detected by LIVE/DEAD cell viability assay. CATH.a cells were either untreated (control) or pretreated with vehicle (MPP/veh), 50 μM LSA (MPP/50) or 100 μM LSA (MPP/100) for 30 min before the stimulation of 600 μM MPP^+^ for 24 hours. The LIVE/DEAD cell viability assay was employed to detect the cell viability. The representative images of the phase contrast (Phase), calcein (green) and EthD-1 (red) are shown in panel (**a**). Panel (**b**) and (**c**) are the fluorescence (arbitrary unit, AU) of calcein and EthD-1, respectively. Results are means ± S.D. from three independent experiments. Significant differences between the cells treated with vehicle and MPP^+^ were indicated by ***, P < 0.001. Significant differences between the cells treated with MPP^+^ combined with vehicle (veh) and LSA were indicated by ###, P < 0.001

For determining the MPP^+^-mediated death pathways of CATH.a cells, calcein/ethidium homodimer-1 staining was employed. Treatment with MPP^+^ elicited both apoptosis and necrosis of CATH.a cells in a concentration-dependent manner (Fig. 3). MPP^+^ (400 μM) did not significantly affect calcein fluorescence (apoptosis), and elicited increase of ethidium homodimer-1 fluorescence (necrosis) by 9.35 folds of control. On the other hand, 600 μM MPP^+^ elicited reduction of calcein fluorescence and increase of ethidium homodimer-1 fluorescence by 39.25 % and 11.58 folds of control, respectively, and 50 μM LSA attenuated those values to 11.40 % and 6.17 folds of control (Fig. 3).

### LSA blocked the MPP^+^-induced accumulation of superoxide anion and decrease of mitochondrial membrane potential

**Fig. 4:**
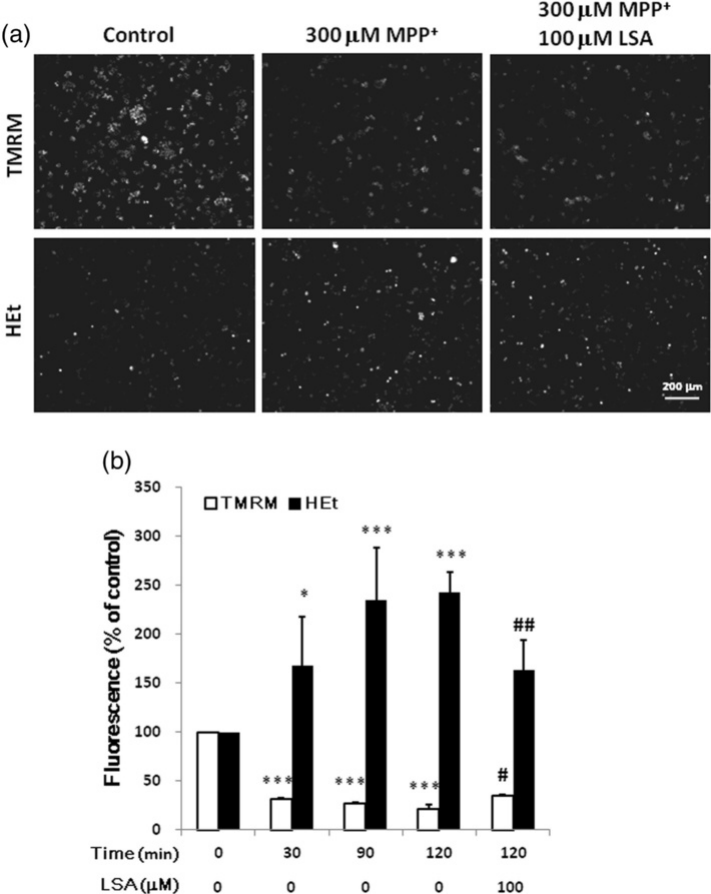
The MPP^+^-induced production of superoxide anion and decrease of mitochondrial membrane potential in CATH.a cells were attenuated by LSA. CATH.a cells were either cultured with 300 μM MPP^+^ for various time-periods or pretreated with 100 μM LSA for 30 min, and then were cultured with 300 μM MPP^+^ for 120 min. After treatments, superoxide anion and mitochondrial membrane potential were detected by the fluorescence of hydroethidine (HEt) and TMRM, respectively. The representative images are shown in panel (**a**). Panel (**b**) is the relative fluorescence (% of control) of TMRM (opened bars) and HEt (closed bars). Results are means ± S.D. from three independent experiments. Significant differences between the cells treated with vehicle and MPP^+^ were indicated by *, P < 0.05, ***, P < 0.001. Significant differences between the cells treated with 300 μM MPP^+^ alone (C) and 300 μM MPP^+^ combined with LSA were indicated by #, P < 0.05; ##, P < 0.01

Dihydroethidium was used to measure the intracellular level of superoxide anion (O_2_^−^.). Treatment with MPP^+^ significantly provoked O_2_^−^. accumulation initially at 30 min and peaked at 120 min (Fig. 4). Treatment with MPP^+^ (300 μM ) for 30, 90, and 120 min elevated the level of O_2_^−^. to 167.3 ± 50.9, 234.9 ± 53.9, and 242.9 ± 20.9 % of control, respectively. LSA (100 μM) diminished the MPP^+^-induced O_2_^−^. accumulation by 55.37 % (Fig. 4b).

TMRM was used to verify whether LSA modulated the mitochondrial membrane potential. Treatment with MPP^+^ significantly diminished mitochondrial membrane potential as soon as at 30 min (Fig. 4). Treatment with 300 μM MPP^+^ for 30, 90, and 120 min decreased the level of membrane potential to 30.93 ± 1.23, 26.45 ± 1.29, and 21.03 ± 4.07 % of control, respectively. LSA (100 μM) significantly recovered the MPP^+^-induced decrease of membrane potential by 17.23 % (Fig. 4).

### LSA Blocked the MPP^+^-Induced Pro-apoptotic responses

**Fig. 5:**
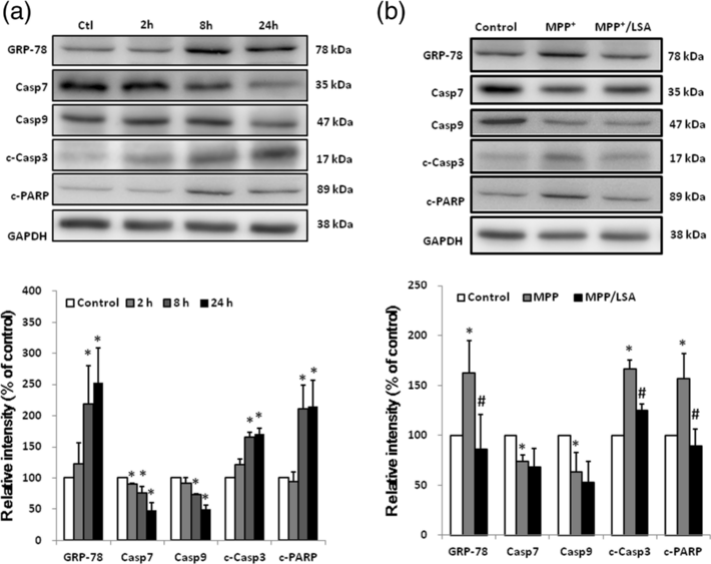
The MPP^+^-induced pro-apoptotic responses in CATH.a cells were attenuated by LSA. CATH.a cells were cultured with 300 μM MPP^+^ for various time-periods (**a**) or pretreated with 100 μM LSA for 30 min, and then were cultured with 300 μM MPP^+^ for 8 h (**b**). After treatments, the indicated proteins were detected by western blotting and the representative images are shown. Lower part of panel (**a**) and (**b**) are the relative protein level of GRP-78, caspase 7 (Casp7), caspase 9 (Casp9), cleaved caspase 3 (c-Casp3) and cleaved PARP (c-PARP). The protein level has been normalized with GAPDH. Results are means ± S.D. from three independent experiments. Significant differences between the cells treated with vehicle and MPP^+^ were indicated by *, P < 0.001. Significant differences between the cells treated with 300 μM MPP^+^ alone and 300 μM MPP^+^ combined with LSA were indicated by #, P < 0.001

MPP^+^ time-dependently induced ER stress in CATH.a cells and increased expression of ER chaperones (GRP-78). MPP^+^ also triggered apoptosis by decreasing the level of the caspase 7 and 9, and increasing the level of cleaved caspase 3 and PARP. Treatment with MPP^+^ (300 μM ) for 24 hour significantly provoked GRP-78, caspase 7, caspase 9, cleaved caspase 3 and cleaved PARP to 252.48 ± 55.90, 48.60 ± 11.87, 49.73 ± 7.34, 170.39 ± 9.12 and 215.28 ± 41.17 of the control, respectively (Fig. 5a). LSA treatment significant prevented the level increase of GRP-78, cleaved caspase 3 and cleaved PARP. Treatment with MPP^+^ (300 μM ) for 8 hour significantly provoked GRP-78, caspase 7, caspase 9, cleaved caspase 3 and cleaved PARP to 162.63 ± 32.50, 73.71 ± 6.32, 63.14 ± 19.45, 166.39 ± 8.88 and 156.78 ± 25.04 of the control, respectively (Fig. 5b). LSA (100 μM) diminished the MPP^+^-induced level increase of GRP-78, cleaved caspase 3 and cleaved PARP to 86.38 ± 34.49, 124.76 ± 6.41 and 89.18 ± 16.99 of the control, respectively (Fig. 5b). Alternatively, LSA did not significantly affected MPP^+^-induced level decrease of caspase 7 and caspase 9. These results indicate that LSA may protect Cath.a cells against MPP^+^ activated ER stress and the associated apoptosis specifically target on caspase 3, but not on caspase 7 and 9.

**Fig. 6:**
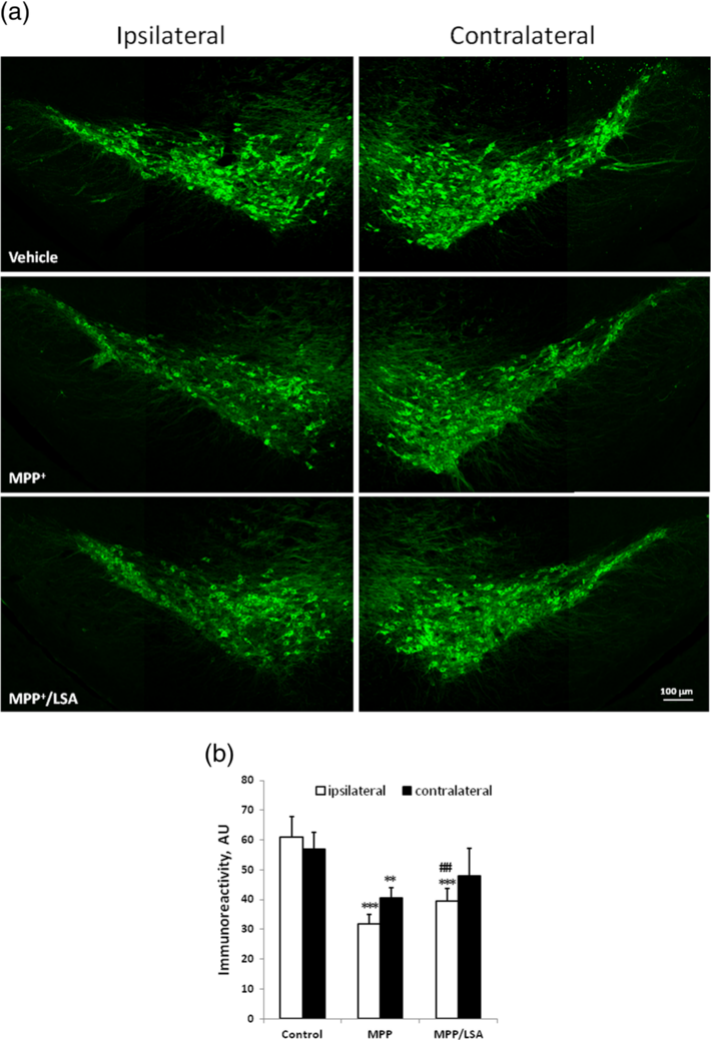
The icv-MPP^+^-injection-induced lost of dopaminergic neurons in SNpc were marginally attenuated by icv-LSA-injection. Two μl vehicle, MPP^+^ (12 μg) alone or MPP^+^ (12 μg) combined with LSA (5 μg) were injected into lateral ventricle of ICR mice. The mice were sacrificed 5 days later. Brain of the mice was subjected to cryosection and immunostained by anti-tyrosine hydrolase antibody (green). Panel (**a**) is the representative fluorescence images. Panel (**b**) is the quantification of tyrosine hydrolase immunoreactivity. Results are means ± S.D. from three independent experiments. Significant differences between the mice treated with vehicle and MPP^+^ were indicated by **, P < 0.01; ***, P < 0.001. Significant differences between the cells treated with 300 μM MPP^+^ alone and 300 μM MPP^+^ combined with LSA were indicated by ##, P < 0.01.

### Icv-MPP^+^-injection-induced lost of dopaminergic neurons in SNpc were attenuated by icv-LSA-injection

To explore the *in vivo* neurotoxicity of MPP^+^ and neuroprotective activity of LSA, the intracerebral ventricular injection of MPP^+^ (icv-MPP^+^-injection, 12 μg in 2 μl saline) alone or MPP^+^ combined with LSA (icv-MPP^+^-LSA-injection, 12 μg and 5 μg, respectively, in saline) were performed at single-side lateral ventricle of ICR mice. The mice were sacrificed 5 days later and the survival of the dopaminergic neurons were detected by immunostaining of TH. The results showed that icv-MPP^+^-injection reduced the TH-immunoreactivity in ipsilateral and contralateral side of SNpc to 51.2 ± 5.1 % and 70.86 ± 6.0 % of the control, respectively (Fig. 6). Co-treatment of LSA with MPP^+^ recovered the lost TH-immunoreactivity in ipsilateral side of SNpc to 64.7 ± 6.9 % and 83.89 ± 16.3 % of the control, respectively.

### Icv-MPP^+^-injection-induced activation of microglia in SNpr and CA1 were attenuated by icv-LSA-injection

**Fig. 7:**
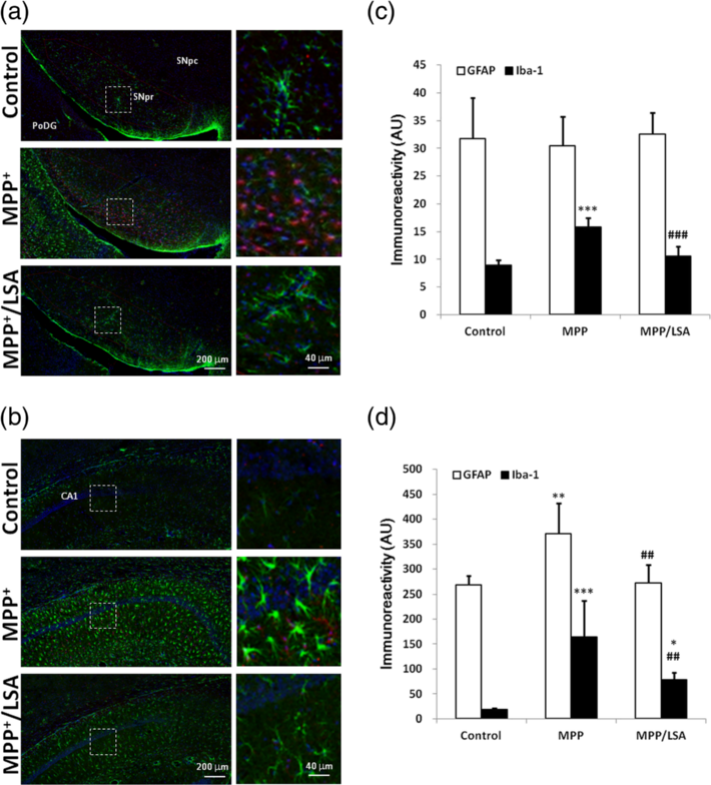
The icv-MPP^+^-injection-induced activation of glial cells in SNpr and CA1 were attenuated by icv-LSA-injection. 2 μl vehicle, MPP^+^ (12 μg) alone or MPP^+^ (12 μg) combined with LSA (5 μg) were injected into lateral ventricle of ICR mice. The mice were sacrificed 5 days later. Brain of the mice was subjected to cryosection and immunostained by anti-GFAP antibody (green) and anti-Iba-1 antibody (red). Panel (**a**) and (**b**) are the representative fluorescence images in SNpr/SNpc/PoDG and CA1 area, respectively. The right part of the panels is the amplified images of the dotted square in the left part of the panel. Panel (**c**) and (**d**) is the quantification of GFAP and Iba-1 immunoreactivity of panel (**a**) and (**b**), respectively. Results are means ± S.D. from three independent experiments. Significant differences between the mice treated with vehicle and MPP^+^ were indicated by *, P < 0.05; **, P < 0.01; ***, P < 0.001. Significant differences between the mice treated with MPP^+^ alone and MPP^+^ combined with LSA were indicated by ##, P < 0.01; ###, P < 0.001

To examine the pro-inflammatory effects of MPP^+^, astrocytes and microglia were detected by immunostained with anti-GFAP and anti-Iba-1 antibodies, respectively. The results showed that icv-MPP^+^-injection increased the GFAP-postive astrocytes in CA1 area (to 138.9 % of the control) but not in SNpc and SNpr. The increased GFAP-positive astrocytes in CA1 area was attenuated by the co-treatment of LSA with MPP^+^ (to 101.5 % of the control). On the other hand, icv-MPP^+^-injection increased the Iba-1-postive microglia in both SNpr and CA1 area to 176 % and 9 folds of the control, respectively (Fig. 7). Co-treatment of LSA with MPP^+^ reduced the Iba-1-postive microglia in SNpr and CA1 area to 118 % and 4.3 folds of the control, respectively.

### Icv-MPP^+^-injection-induced activation of radial glial-like stem cells and new-borne neurons in subgranular zone were attenuated by icv-LSA-injection

**Fig. 8:**
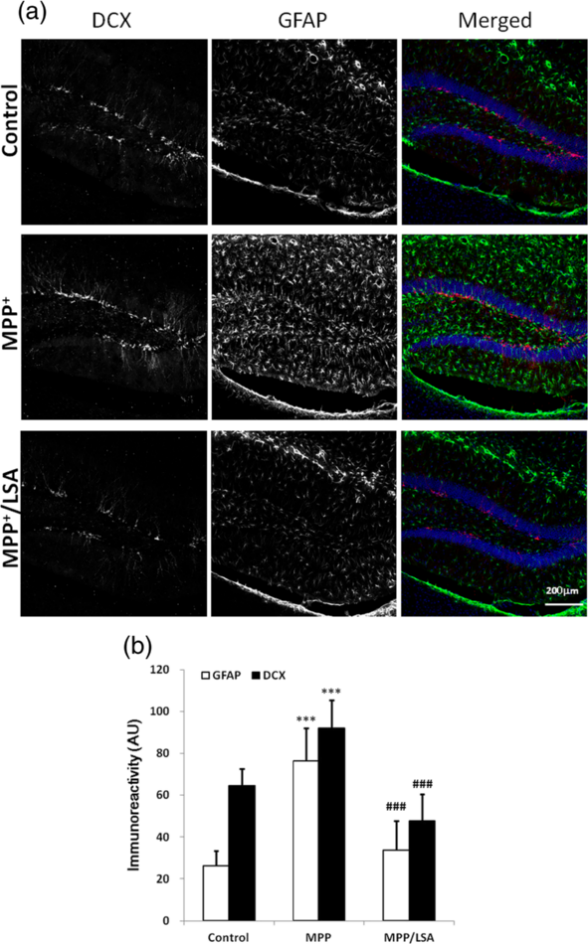
The icv-MPP^+^-injection-induced activation of radial glial-like stem cells and new-borne neurons in subgranular zone were attenuated by icv-LSA-injection. Two μl vehicle, MPP^+^ (12 μg) alone or MPP^+^ (12 μg) combined with LSA (5 μg) were injected into lateral ventricle of ICR mice. The mice were sacrificed 5 days later. Brain of the mice was subjected to cryosection and immunostained by anti-GFAP antibody (green) and anti-doublecortine antibody (DCX, red). Upper panel is the representative fluorescence images. Lower panel is the quantification of GFAP-labeled radial glial-like cells and DCX-positive cells . Results are means ± S.D. from three independent experiments. Significant differences between the mice treated with vehicle and MPP^+^ were indicated by ***, P < 0.001. Significant differences between the mice treated with MPP^+^ alone and MPP^+^ combined with LSA were indicated by ###, P < 0.001

To examine the promoting effects of MPP^+^ on neurogenesis, the radial glial-like stem cells and the new-borne neurons were detected by immunostained with anti-GFAP and anti-doublecortin antibodies, respectively. The results showed that icv-MPP^+^-injection increased the GFAP-positive astrocytes/radial glial-like stem cells and doublecortin-positive new borne neurons in DG area to 291 % and 142 % of the control, respectively (Fig. 8). Co-treatment of LSA with MPP^+^ reduced these value to 128.7 % and 73.3 % folds of the control, respectively.

## Discussion

In the present study, the *in vitro* model (CATH.a cells, a dopaminergic neuron cell line) demonstrated that LSA abrogated MPP^+^-mediated neurotoxicity as determined by MTT reduction, calcein/ethidium homodimer-1 staining, and morphological observation. LSA attenuated the MPP^+^-mediated effects, including accumulation of O_2_^−^., decrease of mitochondrial membrane potential, activation of the caspase cascade, and thereby protected CATH.a cells against MPP^+^-mediated neurotoxicity. Both necrosis and apoptosis are implicated in this neurotoxicity. Moreover, the *in vivo* model (icv-MPP^+^-injection of ICR mice) demonstrated that LSA effectively attenuate MPP^+^-mediated loss of dopaminergic neurons in SNpc, astrogliosis in hippocampus, microglial activation in both SNpr and CA1 areas, and aberrant neurogenesis in hippocampus.

In the *in vitro* assay, LSA and TAB were added into cell culture 30 min before the stimulation of MPP^+^ to prevent those pre-mixed with MPP^+^. In the *in vivo* assay, LSA solution was injected with MPP^+^ solution in the same syringe but without pre-mixed.

Previously, MPP^+^ has been suggested to induce neurotoxicity in dopaminergic neurons [7]. For verifying the above statement, dose response of MPP^+^-mediated neurotoxicity was performed on two different cell types (i.e. CATH.a cells and cortical neurons). CATH.a cells, a mouse brain-derived catecholaminergic neuron cell, synthesizes abundant dopamine and norepinephrine and expresses the appropriate TH and dopamine β-hydroxylase [25]. Previously, CATH.a cells administrated with 200–1000 μM MPP^+^ has been employed to be a cell model for the mechanism studies on Parkinson’s diseases [27]. Besides the dopaminergic neurons, the primary cortical neurons have also been reported to be accessible to MPP^+^-mediated neurotoxicity [28, 29]. In our present study, we use both CATH.a cell line and primary cortical neurons to study the neurotoxic specificity mediated by MPP^+^. Our results indicated that MPP^+^ mediates distinct death pathway in CATH.a cells and the primary cortical neurons although the dose response between these two cell types was similar. The calpain-dependent cleavage of α-spectrin is involved in the death of primary cortical neurons, but not of CATH.a cells. The reason may be due to that CATH.a cells is not a neurite-bearing mature neurons [19, 30] and which may explain why LSA fail to protect the primary cortical neurons against MPP^+^-mediated neuron death. The previous reports suggested that calpain plays a central role in MPP^+^ mediates cell death in spinal cord motoneurons [31] and cerebellar granule neurons [32] may supported this speculation.

Mitochondrion is involved in the activation of caspase 9 mediating by cytochrome *c* release. Therefore, the mitochondrion is a pivotal organelle implicating in apoptosis [33]. Recently, ER has attracted attention as an intracellular compartment in which disturbance of calcium homeostasis may contribute to pathological processes culminating in neuronal injury [34]. Mitochondrial superoxide anion production and membrane potential loss has been reported to be the primitive mechanism of MPP^+^-mediated neuron death [35, 36]. In CATH.a cells, we also find that MPP^+^-induced superoxide anion production and mitochondrial membrane potential loss. These mitochondrial effects were attenuated by LSA treatment suggested that LSA may prevent the primitive mechanism of MPP^+^-mediated neuron death on mitochondria.

MPP^+^-induced toxicity seems to involve activation of unfolding protein response (UPR) and ubiquitin–proteasome system (UPS) dysfunction. In cell lines and primary dopaminergic neurons, MPP^+^ activates UPR; however, which branches are upregulated varies by cell type [10, 11]. Many studies have demonstrated the induction of at least some markers of apoptosis by MPP^+^ [37]. Exactly how MPP^+^ leads to cell death in dopaminergic neurons is still unclear [37]. Many studies have suggested that prolonged and severe UPR can lead to cell death, possibly via apoptosis [38]. Amongst other mechanisms, ER stress pathways have been hypothesized to link to apoptosis [35]. Growing evidence supports that ER could operate in tandem with mitochondria to regulate intracellular Ca^2+^ fluxes in MPP^+^-induced cell death.

The caspase cascade responsible for executing cell death following cytochrome c release is well described; however the distinct roles of caspases-9, −3 and −7 during this process are not completely defined. Caspase-9 can remodel mitochondria and increase ROS production by cleaving Bid into tBid. Also, caspase-3 can inhibit ROS production and is the effector caspase necessary for efficient cell killing. In contrast, caspase-7 has no significant role in sensitivity to intrinsic cell death, but it is responsible for ROS production and cell detachment [39]. Our data showed that mitochondria dependent apoptosis triggered by MPP^+^, assessed by the measurement of caspase 7 and 9 activation, is not prevented by LSA treatment. However, LSA blocks the MPP^+^-mediated increase in GRP-78 expression and caspase 3 activation suggesting that the ER stress but not the mitochondrial pathway may be regulated by LSA. In the previous studies, the protein levels of GRP-78 in the MN9D cells were up-regulated by 6-OHDA or MPP^+^ [11], suggesting that ER stress is indeed a major pathway of MPP^+^-mediated neuronal apoptosis or a survival effect to save cells. Inhibition of complex I in mitochondria by MPP^+^ triggers generation of ROS and RNS. As a result of close proximity of mitochondria and ER, critical thiols of IP_3_R are modified by mitochondrial ROS and RNS. Consequently, IP_3_R channels are activated thereby enhancing ER Ca^2+^ release, calpain activation and Bax cleavage. Cleavage of Bax facilitates Bax oligomerization and formation of death pores in the outer mitochondrial membrane that leaks cytochrome c into the cytosol which, in turn, leads to apoptosome formation followed by caspase-9/caspase-3 activation and apoptosis.

The ip-injection of MPTP is the most relevant animal model of PD. On the other hand, to bypass the blood–brain barrier, MPTP is injected stereotactically into the brain. However, icv-injected MPTP induced no lesions in the striatum or SNpc of C57BL/6 mice [40]. It is possible that icv-injected MPTP rapidly disappears from CSF before the conversion to MPP^+^ and/or that MPTP is detoxified by drug-metabolizing enzymes such as glutathione S-transferase and cytochrome P450. Therefore, icv-injection of MPP^+^ may be used to prohibit this disadvantage. After icv injection, MPP^+^ initially affects the dopaminergic cell body in the SNpc and then the striatum to which dopaminergic cells project [40].

Previously, several polyphenols and polyphenol glycosides have been demonstrated to be neuroprotective against MPP^+^-mediated neurotoxicity that include puerarin [41]; tetrahydroxystilbene glucoside [42]; pedicularioside A [43]; myricetin [38]; pinocembrin [44]; daidzein [45]; acacetin [46]; quercetin [47]. Moreover, salvianolic acid (Sal) B and A, were also found to protect SH-SY5Y cells against MPP^+^-induced apoptosis by relieving oxidative stress and modulating the apoptotic process [48, 49]. The structure of LSA is similar to Sal B and A. In acidic aqueous solution, Sal B are found to be hydrolyzed into LSA, and the latter was transformed into Sal A [50]. In our present study, LSA was found to protect CATH.a cells against MPP^+^-induced apoptosis by relieving oxidative stress and modulating the apoptotic process.

## Conclusion

In conclusion, LSA is potential for novel therapeutic intervention for Parkinson’s disease.

## Abbreviations

BrdU: 5-bromo-2’-deoxyuridine
CA: Cornu Ammonis
DG: Dentate dyrus
ER: Endoplasmic reticulum
GFAP: Glial fibrillary acid protein
GRB-78: 78 kDa glucose-regulated protein
Iba-1: Ionized calcium-binding adaptor molecule-1
icv: Intracerebral ventricle
IR: Immunoreactivity
JNK: C-jun N-terminal kinase
LSA: Lithospermic acid
MPP^+^: 1-methyl-4-phenylpyridine
MPTP: 1-methyl-4-phenyl-1,2,3,6- tetrahydropyridine
MTT: 3-[4,5-dimethylthiazol-2-yl]- 2-5-diphenyl-tetrazolium bromide
PARP: Poly ADP ribose polymerase
PBS: Phosphate-buffered saline
PD: Parkinson’s disease
RNS: Reactive nitotrogen species
ROS: Reactive oxygen species
SN: Substantia nigra
SNpc: SN pars compacta
SNpr: SN pars reticulata
TAB: Tournefolic acid B
TABM: Tournefolic acid B methyl ester
TH: Tyrosine hydrolase
TMRB: Tetramethyl Rhodamine Methyl Ester
UPR: Unfolding protein response
UPS: Ubiquitin–proteasome system
